# Localization of RalB signaling at endomembrane compartments and its modulation by autophagy

**DOI:** 10.1038/s41598-019-45443-1

**Published:** 2019-06-20

**Authors:** Manish Kumar Singh, Alexandre P. J. Martin, Carine Joffre, Giulia Zago, Jacques Camonis, Mathieu Coppey, Maria Carla Parrini

**Affiliations:** 1 Institut Curie, Centre de Recherche, Paris Sciences et Lettres Research University, 75005 Paris, France; 2 ART group, Inserm U830, 75005 Paris, France; 3 Centre de Recherches en Cancérologie de Toulouse (CRCT), Inserm UMR1037, Toulouse, France; 4 LOCCO group, UMR168, 75005 Paris, France

**Keywords:** Autophagy, Imaging, Cell signalling

## Abstract

The monomeric GTPase RalB controls crucial physiological processes, including autophagy and invasion, but it still remains unclear how this multi-functionality is achieved. Previously, we reported that the RalGEF (Guanine nucleotide Exchange Factor) RGL2 binds and activates RalB to promote invasion. Here we show that RGL2, a major activator of RalB, is also required for autophagy. Using a novel automated image analysis method, Endomapper, we quantified the endogenous localization of the RGL2 activator and its substrate RalB at different endomembrane compartments, in an isogenic normal and Ras-transformed cell model. In both normal and Ras-transformed cells, we observed that RGL2 and RalB substantially localize at early and recycling endosomes, and to lesser extent at autophagosomes, but not at trans-Golgi. Interestingly the use of a FRET-based RalB biosensor indicated that RalB signaling is active at these endomembrane compartments at basal level in rich medium. Furthermore, induction of autophagy by nutrient starvation led to a considerable reduction of early and recycling endosomes, in contrast to the expected increase of autophagosomes, in both normal and Ras-transformed cells. However, autophagy mildly affected relative abundances of both RGL2 and RalB at early and recycling endosomes, and at autophagosomes. Interestingly, RalB activity increased at autophagosomes upon starvation in normal cells. These results suggest that the contribution of endosome membranes (carrying RGL2 and RalB molecules) increases total pool of RGL2-RalB at autophagosome forming compartments and might contribute to amplify RalB signaling to support autophagy.

## Introduction

The two human Ral proteins (RalA and RalB) are monomeric GTPases which are activated by RalGEFs (Guanine Nucleotide Exchange Factors)^1,2^. Among the six identified RalGEFs, four contain a Ras-association (RA) domain (RGL1, RGL2, RGL3, RalGDS) and are direct effectors of Ras GTPases oncogene proteins (K-Ras, H-Ras, N-Ras). The Ral pathway is permissive if not instructive per se for the Ras induced oncogenesis^3^ and for autophagy^4^.

Very frequent oncogenic events in human cancers are mutations of Ras oncoproteins resulting in their constitutive activation^5^. Hence, Ral signaling is a potential target for anti-cancer therapeutic strategy, which is yet not exploited^6^. The studies on Ral signaling network led to the discovery of an impressive variety of cellular functions which are under the control of Ral proteins, such as motility and invasion^7–12^, membrane trafficking^13–15^, autophagy^4,16–18^, apoptosis^19,20^, and cell division^21,22^. Intriguingly, even though in some cellular contexts RalA and RalB seem to have overlapping effects, a distinct role for RalB activity was reported in specifically regulating two important cellular processes: motility/invasion^7–9,11,23–25^ and autophagy^4,26^. How RalB coordinates the interplay between invasion and autophagy, particularly in the context of cancer cells with Ras mutations, remains unanswered.

One possible explanation for this functional versatility could be that RalB is activated at specific sub-cellular locations^27,28^, by distinct RalGEFs, with specific temporal features. The notion that activated GTP-bound Ras recruits RalGEFs at the plasma-membrane, triggering the activation of RalB by GDP/GTP exchange, is well supported by experimental evidences^25,29^, however, the possibility of Ras to Ral signaling occurring at endomembranes remains poorly explored, partially because of technical difficulties.

When studying protein localizations at endomembrane compartments (such as endosomes, autophagosomes, Golgi apparatus), the existing analysis approaches present several drawbacks. The most common approach is to compose an overlay image of dual color images (e.g. green and red): the presence of both green and red biomolecule at same pixels results in yellow spots^30,31^. However, since the subsequent yellow spots totally depend on the signal strength measured in green and red channels, the approach is reliable only if both channels show similar grey level dynamics. Second commonly used approach is based on the cross-correlation analysis of grey value of dual channel images (e.g. Pearson correlation coefficient and the Mander’s overlap coefficient)^32,33^. But, these coefficients rely on signal proportionality of two probes, which can be misleading if the probe ratio varies widely^31^. Moreover, in addition to localization, it is also important to measure the local activities of the proteins of interest. For this work, we developed a robust automated method in order to quantify the endomembrane compartments that are positive for proteins of interest. We named this method “Endomapper”, for Endomembrane mapping of proteins of interest. This method is independent of probe signal strength or its proportionality because it uses one channel to segment endomembrane compartments and another to measure protein intensity.

We applied the “Endomapper” to the study of RalB localization and activity at endomembranes, specifically at early endosomes (identified by EEA1 and Rab5), recycling endosomes (identified by Rab11), autophagosomes (identified by LC3), and trans-Golgi (identified by Rab6). This image analysis approach revealed the presence of both RalB and its activator RGL2 at early and recycling endosomes, and to a less extent at autophagosomes. Further, we characterized how starvation-induced autophagy affects RGL2/RalB localization and RalB activity at endomembrane compartments.

## Results

### RGL1 and RGL2 are key activators of RalB for both invasion and autophagy

**Figure 1:**
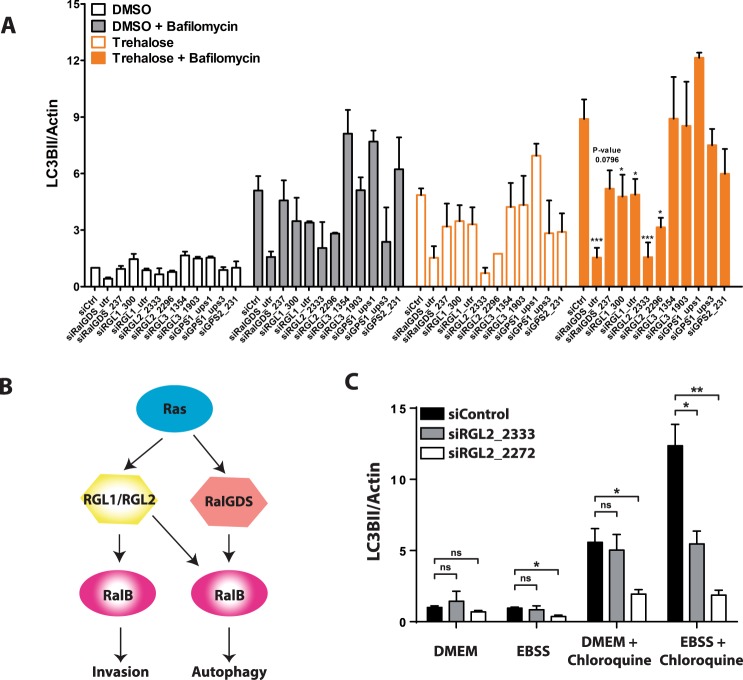
Role of RalGEFs in autophagy. (**A**) The RalGEFs’ screen for autophagy. RalGDS, RGL1, and RGL2 are required for autophagy. Hela cells were depleted by siRNAs against each of the six RalGEFs. Trehalose (100 mM for 16 hour) was used to induce autophagy. Bafilomycin (200 nM) was used to block autophagy flux. The LC3II/actin ratios were calculated and normalized for siControl condition. Graphs show the mean +/− SEM, from 2–3 experiments per condition. For statistics one-way ANOVA followed by Dunnett’s Multiple Comparison test was used to compare the siRNA RalGEF effects with the siControl of same culture condition. *p < 0.05, **p < 0.01, ***p < 0.001. (**B**) RGL1 and RGL2 act down-stream Ras to activate RalB for both invasion and autophagy. (**C**) RGL2 is required for autophagy in HEK-HT cells. HEK-HT cells were transiently transfected with indicated siRNAs. Nutrient-deprived medium (EBSS) for 4 hrs was used to induce autophagy. Chloroquine (50 µM) was used to block autophagy flux. Graphs show the mean +/− SEM, from 3 independent experiments per condition. For statistics Student’s t test was used. *p < 0.05, **p < 0.01, ***p < 0.001, ns not-significant.

We previously reported that, among the six RalGEFs, RGL1 and RGL2 are required to activate RalB for promoting invasion down-stream oncogenic Ras^25^. Since autophagy is another process regulated by RalB^4,26^, we aimed at identifying the specific RalGEF(s) required for autophagy. siRNA mediated depletion of all six RalGEFs was performed in Hela cells and autophagy was followed by LC3 conversion assay^34^. Depletion of RalGDS, RGL1, and RGL2 impaired autophagy (Figs 1A, S1A). Thus RGL1 and RGL2, which act down-stream Ras since they have a Ras-association domain (RA), are key activators of RalB for both invasion and autophagy (Fig. 1B). On contrary RalGDS, another Ras-dependent RalGEF, is required for autophagy, as previously shown in a mouse model^35^, but dispensable for invasion^25^.

For the rest of this study we focused our attention on RGL2 because of its dual function in autophagy and invasion, and because a function for RGL2 had been previously reported at endosomes^36^.

To evaluate the contribution of oncogenic Ras in the activation of RGL2-RalB signaling, we used a genetically controlled cell model for the rest of the study: the HEK-HT cells, which are immortalized but not transformed, and become tumorigenic, invasive and metastatic upon expression of constitutive active H-RasV12^37,38^. Moreover, because of their flat morphology, these cells are suitable for imaging studies. We confirmed the requirement of RGL2 for autophagy in this cell model: RGL2 silencing with two independent siRNAs impaired starvation-induced autophagy in HEK-HT cells, as assessed by LC3 conversion assay (Figs 1C, S1B).

### An automated image analysis method to quantify localization of proteins of interest at endomembrane compartments

**Figure 2:**
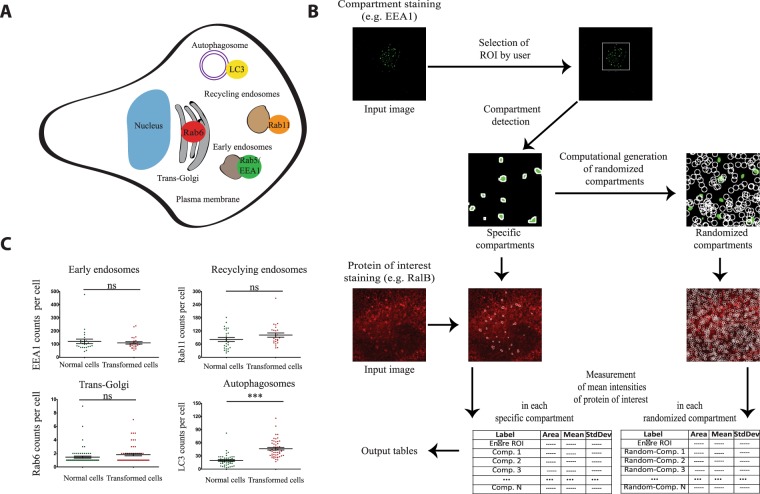
An automated image analysis method to quantify localization of proteins of interest at endomembrane compartments. (**A**) The endomembrane compartments. Schematic representation of the different endomembrane compartments investigated in this study and their markers. (**B**) Work-flow of the Endomapper ImageJ plug-in. (**C**) Counts of different endomembrane compartments in HEK-HT (normal cells) and HEK-HT-H-RasV12 (transformed cells). Graph represents mean ± SEM of n > 35 cells from 4 independent experiments. Each dot corresponds to one cell. For statistics Mann Whitney test was used. **p < 0.01, ***p < 0.001, ns not-significant.

In order to quantify the localization of RGL2 and RalB at endomembranes (Fig. 2A), we developed a novel automated image analysis method, named “Endomapper” (Fig. 2B).

In a first step, Endomapper uses one channel to identify specific endomembrane compartments (for example early endosomes, using EEA1 marker) and another channel to measure the mean intensity of the protein of interest (for example RalB, using specific anti-RalB antibodies) within these compartments. In a second step, in order to correct for co-localization by chance, Endomapper creates “randomized” pseudo-compartments whose number and size can be modulated by user. For example, we used n = 1000 and area = 1.2 μm^2^ for endosomes and autophagosomes, and n = 10 and area = 9.2 μm^2^ for trans-Golgi, to mimic the properties of the specific compartments under investigation. Since the localization of the pseudo-compartments is random, they could colocalize with the real endomembrane compartments by chance. The assumption is that a protein of interest is really localized at particular endomembrane compartments only if there is a significant difference between the measurements at the specific compartments as compared to the measurements at the pseudo-compartments. The region of interest for the analysis is defined by the user in a way to restrict the analysis to cell area where the specific compartments are present.

We tested various thresholds to identify a good compromise between specificity and sensitivity (Fig. S3). We chose to consider an endomembrane compartment positive for a protein of interest only if its mean intensity was above the sum of mean intensity (of all compartments) and standard deviation (mean +1 fold SD) within the region of interest. As benchmark, we assessed this Endomapper method by quantifying the co-localization of two different markers for the same compartment: the EEA1 and Rab5 markers of early endosomes. Almost 100% of EEA1 compartments were positive for Rab5, both in normal and Ras-transformed cells (Fig. S4), confirming that the method is efficient and robust.

Endomapper was also useful to simply count the number of specific endomembrane compartments per cell (Fig. 2C). No differences in the counts per cell of early endosomes, recycling endosomes and Trans-Golgi compartments in HEK-HT versus HEK-HT-H-RasV12 cells were observed, indicating that oncogenic Ras does not globally impact on the organization of these endomembranes at basal level. Interestingly, we observed 2.3 fold more autophagosomes (LC3-positive compartments) in Ras-transformed cells than in isogenic normal cells, in agreement with previous reports on increased autophagy in Ras-driven cancer cells^39,40^.

### RGL2 and RalB localization at early endosomes, recycling endosomes, and autophagosomes

**Figure 3:**
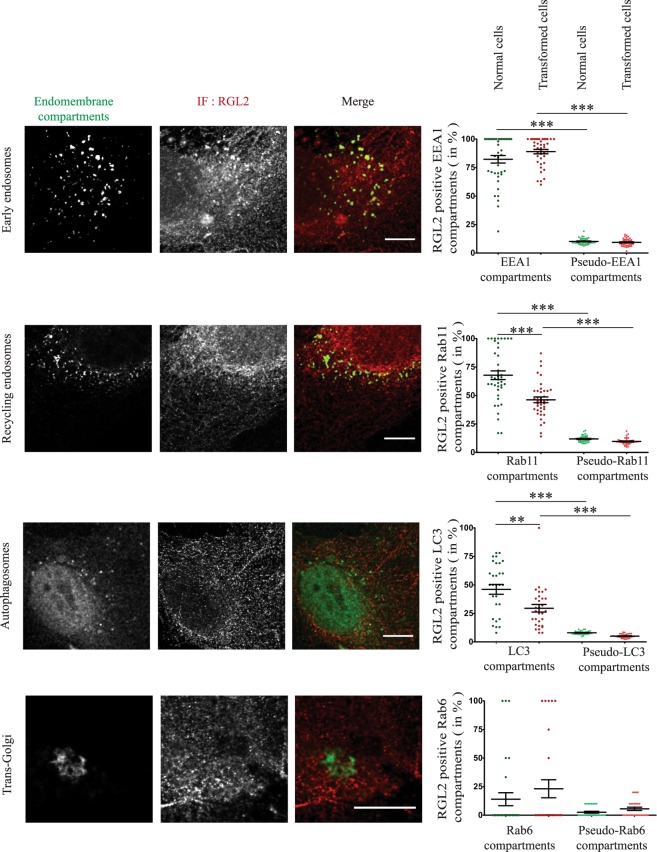
Localization of RGL2 at different endomembrane compartments. HEK-HT (Normal cells) and HEK-HT-H-RasV12 (Transformed cells) cells were fixed and imaged for endogenous RGL2 (IF anti-RGL2, represented in red), together with EEA1 (early endosome marker, IF anti-EEA1, represented in green), GFP-Rab11 (recycling endosome marker, represented in green), GFP-Rab6 (Trans-Golgi marker, represented in green), or iRFP-LC3 (autophagosome marker, represented in green). Representative confocal cross sections of normal cells are shown (left). Quantifications are reported for both normal and transformed cells (right). Localization was calculated as percentage (%) of the indicated endomembrane compartments positive for RGL2, as compared to the control pseudo-compartments. Each dot corresponds to one cell. Graph represents mean ± SEM of 21 to 40 cells from 3–4 independent experiments. For statistics Mann Whitney test was used. **p value < 0.01, ***p value < 0.001. Scale bars are 10 µm.

The Endomapper method revealed that a substantial fraction of early endosomes (EEA1 compartments) and recycling endosomes (Rab11 compartments) was positive for endogenous RGL2 (~80% and ~60%, respectively). RGL2 was also found at ~30% of autophagosomes (LC3 compartments). In contrast, RGL2 was not present at Trans-Golgi (Rab6 compartments) since no significant difference was found between the measurements at Rab6 specific compartments as compared to the measurements at the pseudo-compartments (Fig. 3). A significant decrease of RGL2 was observed at recycling endosomes and autophagosomes in Ras-transformed cells as compared to normal cells. One possibility is that RGL2 molecules are re-localized by Ras-GTP from endomembranes to plasma-membrane during Ras-dependent transformation^25^.

**Figure 4:**
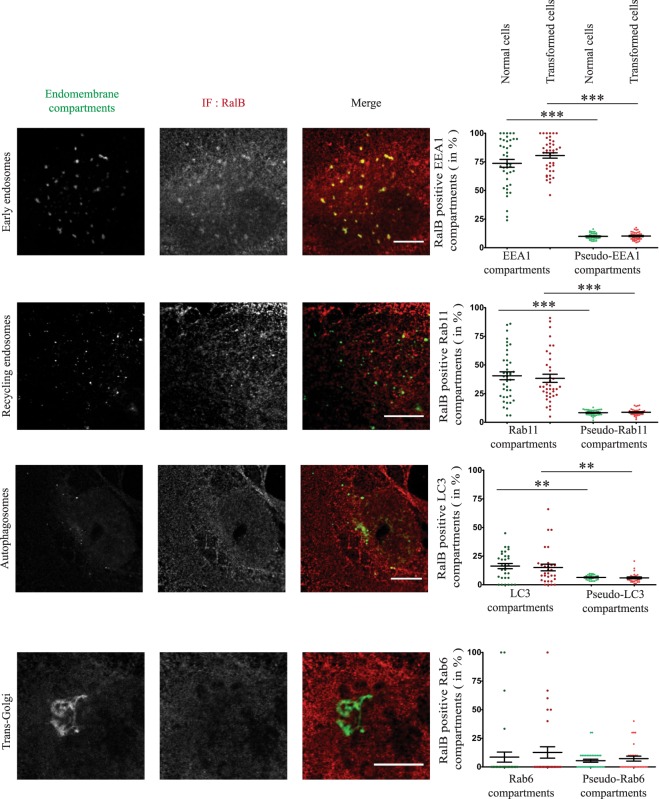
Localization of RalB at different endomembrane compartments. HEK-HT (Normal cells) and HEK-HT-H-RasV12 (Transformed cells) cells were fixed and imaged for endogenous RalB (IF anti-RalB, represented in red in overlay images), together with EEA1 (early endosome marker, IF anti-EEA1, represented in green), GFP-Rab11 (recycling endosome marker, represented in green), GFP-Rab6 (Trans-Golgi marker, represented in green), or iRFP-LC3 (autophagosome marker, represented in green). Representative confocal cross sections of normal cells are shown (left). Quantifications are reported for both normal and transformed cells (right). Localization was calculated as percentage (%) of the indicated endomembrane compartments positive for RalB, as compared to control pseudo-compartments. Each dot corresponds to one cell. Graph represents mean ± SEM of 21 to 40 cells from 3–4 independent experiments. For statistics Mann Whitney test was used. **p value < 0.01, ***p value < 0.001. Scale bars are 10 µm.

The localization of endogenous RalB was very similar to that of RGL2. A substantial fraction of early endosomes (EEA1 compartments) and recycling endosomes (Rab11 compartments) was positive for endogenous RalB (~75% and ~40%, respectively). RalB was also present at ~20% of autophagosomes (LC3 compartments). In contrast, RalB was not present at Trans-Golgi (Rab6 compartments) (Fig. 4). Also, we did not notice any detectable differences between normal and transformed cells concerning the RalB localization at endomembrane compartments. Interestingly, RalA was also present at ~20% of autophagosomes (Fig. S5), even though it is well established that RalA does not regulate autophagy^4^, thus confirming that localization and function should be evaluated independently.

In conclusion, both RGL2 and RalB localize at early and recycling endosomes, and to lesser extent at basal autophagosomes (in rich medium), suggesting the potential existence of an active RGL2-RalB signaling axis at these subcellular localizations.

### Modulation of endomembrane compartments and RGL2/RalB localization upon autophagy

**Figure 5:**
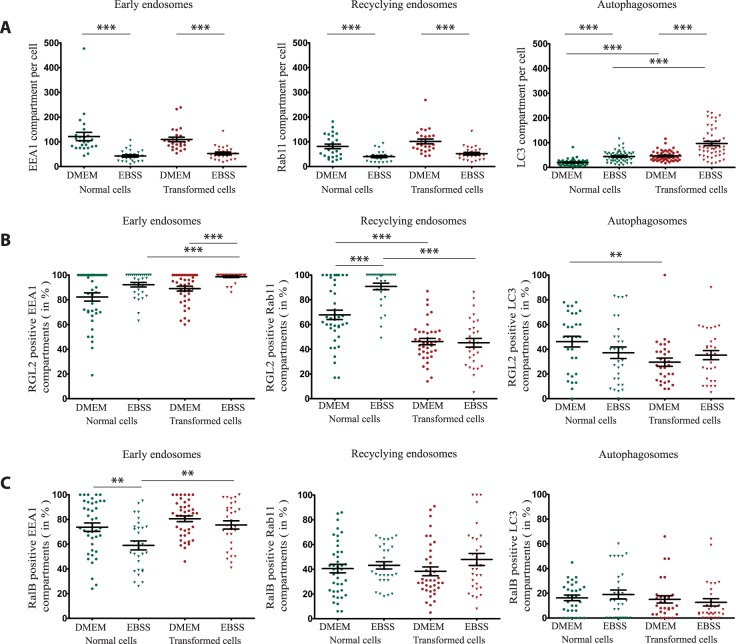
Modulation by autophagy of endomembrane compartments and of RGL2 and RalB localization. (**A**) Modulation by autophagy of endomembrane compartments. The different endomembrane compartments were counted in HEK-HT (Normal cells) and HEK-HT-H-RasV12 (Transformed cells), in basal (DMEM) and starvation (EBSS) condition. To allow comparison DMEM versus EBSS, the same counts in basal conditions of Fig. 2C were reported also here. (**B**) Modulation by autophagy of RGL2 localization at endomembranes. Normal and transformed cells were incubated in either basal (DMEM) or starvation (EBSS) medium (4 hours) before fixation. To allow comparison DMEM versus EBSS, the same measurements in basal conditions of Fig. 3 were reported also here. (**C**) Modulation by autophagy of RalB localization at endomembranes. Normal and transformed cells were incubated in either basal (DMEM) or starvation (EBSS) medium (4 hours) before fixation. To allow comparison DMEM versus EBSS, the same measurements in basal conditions of Fig. 4 were reported also here. Graph represents mean ± SEM of 21 to 40 cells from 4 independent experiments. For statistics Mann Whitney test was used. ** p value < 0.01, ***p value < 0.001.

To characterize the impact of starvation-induced autophagy on RGL2/RalB localization at endomembranes, normal HEK-HT cells and transformed HEK-HT-H-RasV12 cells were grown in basal rich (DMEM) medium or starvation (EBSS) medium for 4 hours to induce autophagy. The counts of the compartments (Fig. 5A) showed an increase of autophagosomes on starvation, as expected, in both normal and transformed cells. Interestingly, a substantial reduction of early and recycling endosomes was observed upon starvation, supporting the notion that maturation of autophagosomes involves fusion with endosomes to contribute in formation of amphisomes which eventually will fuse with lysosomes^41^.

However, percent distributions of both RGL2 (Fig. 5B) and RalB (Fig. 5C) within compartments were overall mildly affected by autophagy, with only two noticeable effects: autophagy induction stimulated RGL2 association to recycling endosomes in normal cells, but not in Ras-transformed cells; RalB dissociated from early endosomes during autophagy in normal cells, but not in Ras-transformed cells. As consequence, upon autophagy, RGL2 and RalB were more present at early endosomes, and RGL2 was less present at recycling endosomes and autophagosomes, in HEK-HT-H-RasV12 cells as compared to normal HEK-HT cells. Taken together, these observations show that autophagy induces a profound reorganization of endomembranes, as expected, and subtle changes in RGL2 and RalB localizations which potentially could contribute to the autophagy process.

### RalB activation at endomembranes and its modulation by autophagy

**Figure 6:**
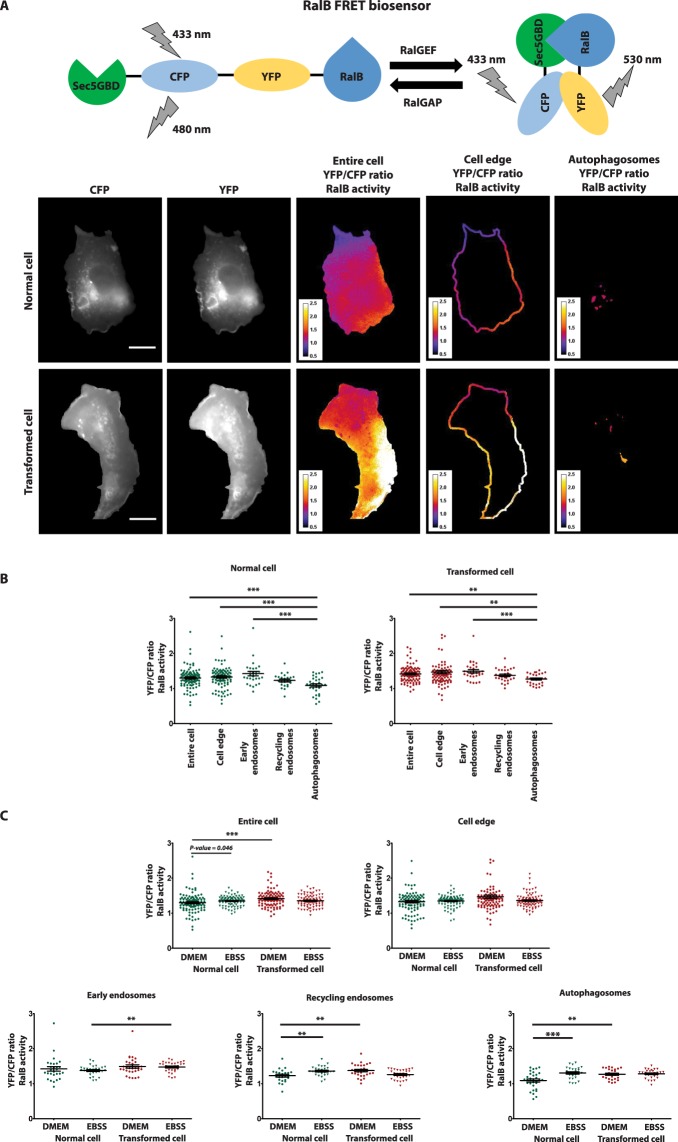
Localization of RalB activity at different cellular compartments and its modulation by autophagy. (**A**) Measurement of local RalB activities using a FRET-based biosensor. Representative HEK-HT and HEK-HT-H-RasV12 cells expressing a RalB FRET biosensor are shown in basal condition. FRET, measured by calculating the ratio of YFP to CFP intensity after subtracting background, is used as indicator of RalB activation at various subcellular locations. Representative ratiometric images (YFP/CFP) are represented with a color code for entire cell, cell edge (1.67 μm–wide band) and autophagosomes. (**B**) Comparison of RalB activity at various subcellular localizations, in HEK-HT cells and HEK-HT-H-RasV12 cells. Measurements of FRET ratio in normal HEK-HT and transformed HEK-HT-H-RasV12 cells in basal condition at different cellular compartments: entire cell, cell edges, early endosomes, recycling endosomes, and autophagosomes. Each dot corresponds to one cell. (**C**) Modulation by autophagy of local RalB activities. Normal and transformed cells were incubated in either basal (DMEM) or starvation (EBSS) medium (4 hours) before imaging. To allow comparison DMEM versus EBSS, the same measurements in basal conditions of panel 6B were reported also here. Graph represents mean ± SEM of n = 26 to 37 cells for entire cell and cell edges, and n = 7 to 10 cells for each endomembrane compartment, from 3 independent experiments. For statistics Mann Whitney test was used, **p < 0.01, ***, and p < 0.001. Scale bars are 20 µm.

In order to assess the activation status of RalB molecules at endomembranes, we used a FRET-based RalB biosensor^16^ that monitors the balance of RalGEF (Guanine Nucleotide Exchange Factor) and RalGAP (GTPase Activating Proteins) activities. We measured RalB activity in the entire cell and at selected cellular localizations: at cell edge (1.67 μm-wide band, mainly reflecting activity at plasma-membrane), at early endosomes, recycling endosomes, and autophagosomes, segmented as previously with specific markers (RFP-Rab5A, mCherry-Rab11, and mCherry-LC3) (Fig. 6).

Interestingly, in rich basal medium, RalB was equally activated at cell edge (i.e. plasma-membrane) and at endosomes, but less activated at autophagosomes, in both normal and transformed cells (Fig. 6B).

Ras-transformed HEK-HT-H-RasV12 cells have been previously reported to have higher RalB-GTP levels than parental HEK-HT cells by biochemical pull-down assay^42^. Consistently, the FRET-based RalB biosensor monitored higher RalB activity in entire HEK-HT-H-RasV12 cells, and also locally at autophagosomes, as compared with HEK-HT cells (Fig. 6C).

When autophagy was induced by starvation (in EBSS medium), we observed a substantial increase of RalB activity at autophagosomes in normal HEK-HT cells, but not in transformed HEK-HT-H-RasV12 cells, probably because in these cells RalB activity was already high at basal level, maybe saturated. Consistently with previous biochemical pull-down assay on whole cell lysates^4^ we observed by FRET in HEK-HT cells an increase of RalB activity upon starvation also at entire cell level (p value = 0.046) (Fig. 6C). No changes were observed upon starvation for the other subcellular localizations, with the exception of a significant increase of RalB at recycling endosomes in normal cells (Fig. 6C). Taken together, these results support the relevance of keeping RalB active in a spatio-temporal manner, specifically at autophagosomes, during the autophagy process.

**Figure 7:**
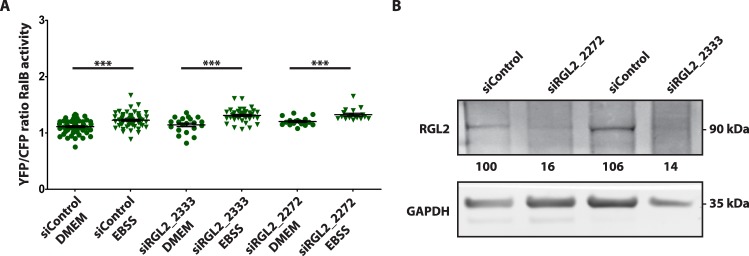
Impact of RGL2 depletion on RalB activation at autophagosomes upon starvation. (**A**) Modulation by autophagy of RalB activity at autophagosomes upon RGL2 depletion. Normal HEK-HT cells were transfected with indicated siRNAs (day 0), then transfected with FRET RalB biosensor and Cherry-LC3 plasmids (day 2), and incubated in either basal (DMEM) or starvation (EBSS) medium (4 hours) before imaging (day 3). Graph represents mean ± SEM of n = 17 to 57 cells per condition, from 3 independent experiments. To compare DMEM versus EBSS conditions Mann Whitney test was used, **p < 0.01, ***, and p < 0.001. (**B**) Validation of RGL2 depletions. Representative western blots for RGL2 and GAPDH from cell lysates of HEK-HT cells prepared 72 hrs after transfection with the indicated siRNAs. Quantifications of RGL2 protein depletion, normalized for siControl condition (=100), are shown below the WB.

Next we addressed the question whether the activation of RalB at autophagosomes during autophagy requires RGL2, since this RalGEF is necessary for autophagy and is localized at autophagosomes. In HEK-HT cells we silenced RGL2 with two independent siRNAs and we measured RalB activity by FRET at autophagosomes, segmented with the mCherry-LC3 marker. Silencing of RGL2 did not impair RalB activation at autophagosomes during starvation (Fig. 7), indicating that RGL2 is not the major regulator of local RalB activity at autophagosomes during autophagy.

## Discussion

This study reveals that, beyond the expected signaling at the plasma-membrane, a signaling path might also operate from Ras to RalB, via RGL2 and other RalGEFs, at endomembranes. A detailed spatial quantification of this intracellular signaling was possible by exploiting the novel “Endomapper” method to evaluate protein localizations at endomembrane compartments. The main advantage of Endomapper over the existing methods is that it quantifies the localization of a protein of interest at specific compartments with respect to randomly generated pseudo-compartments, correcting for co-localization occurring by chance and thus allowing robust statistics even on not obvious images. Moreover, the analysis task is fully automated by the use of a biologist-friendly free-access plugin with the ImageJ software.

Our finding that both RGL2 and RalB substantially localize at early endosomes, recycling endosomes, and autophagosomes, suggests the existence of an active RGL2-RalB signaling axis at these subcellular localizations. However, since it was not technically feasible to simultaneously stain these two proteins, we cannot exclude the possibility that they are on separate pools of endosomes and autophagosomes. A previous study showed that exogenous RGL2 localized at endosomes, where it could activate RalA to promote exocytosis^36^; however, the implication of RalB was not explored.

Further, we observed that starvation-induced autophagy leads to a substantial increase of RalB activity at autophagosomes in normal HEK-HT cells, as assessed by a FRET-based RalB biosensor. On the other hand, the increase of autophagosomes number upon starvation was associated with a considerable reduction of early and recycling endosomes, while the association of both RGL2 and RalB to the various endomembranes was only mildly affected. The biogenesis of autophagosomes is very complex and clearly involves contributions from several membrane compartments^43,44^. We speculate that the fusion of endosomes (carrying RGL2 and RalB molecules) with autophagosome forming compartments might be sufficient to locally increase RGL2-RalB pool at autophagosomes and could contribute to amplify RalB signaling to support autophagy.

In conclusion, this works shows that RalB signaling occurs not only at the plasma-membrane but also at endomembranes. We previously demonstrated that activation of RalB by RGL2 at plasma-membrane leads to protrusions and invasiveness^25^. Here, we show the requirement of RGL2 for autophagy and the presence of both RGL2 and RalB at autophagosomes. However, RGL2 depletion does not reduce RalB activity at autophagosomes during starvation, not supporting a simple model of a unique RGL2-RalB signalling axis mobilized at autophagosomes, and suggesting the involvement of others RalGEFs (such as RalGDS and/or RGL1, identified in the original screen) or RalGAPs. It has been proposed that RalB acts at the very beginning of the process needed to initiate autophagosome formation^4^, so we cannot exclude the possibility that RalB activation (by RGL2 and/or other RalGEFs) is required before association of RalB with LC3+ autophagosome. If so, what is RGL2 doing there remains elusive. Similarly, activation of RalB by RGL2 at endosomes might participate to other functions that need further investigation. This complex spatio-temporal control of RalB localization and its activation is likely the key to understand its functional versatility.

## Materials and Methods

### Plasmids, siRNAs, reagents, antibodies

Lists of plasmids (Table 1), siRNAs (Table 2), reagents (Table 3), and antibodies (Table 4) are provided below.

**Table 1:** List of plasmids used in this study.

pCherry-Rab6 (Bruno Goud lab, Institut Curie)
pRFP-Rab5A (Bruno Goud lab, Institut Curie)
pEGFP-Rab11A (Clontech pEGFP-C1 vector backbone) (Bruno Goud lab, Institut Curie)
pCDNA3-RalB FRET Biosensor^33^
pCherry-LC3 (Patrice Codogno lab)
pLVXW-iRFP-LC3B (this work)
pCherry-Rab11 (Clontech pEGFP-C1 vector backbone) (Bruno Goud lab, Institut Curie)
pIRES-Hygro-Cherry-Rab11A (Bruno Goud lab, Institut Curie)

**Table 2:** List of siRNA sequences used in this study.

siRNA name	Target sense sequence
siControl	ON-TARGET plus Non-targeting siRNA #1 (Dharmacon)
siRalGDS_utr	5′-AACCAGAGGACUAGCUGACUU-3′
siRalGDS_237	5′-CGGGAACCGAAGCACGAAATT-3′
siRGL1_300	5′-CCAUAAUACAGCUCCUAAATT-3′
siRGL1_utr	5′-AACAGUGAUUGUCCCGUUAAU-3′
siRGL2_2296	5′-GGAUGGAGCUUCACACGAUTT-3′
siRGL2_2272	5′-GCUAAUGUAUUCUACGCCATT-3′
siRGL2_2333	5′-CGAAGGUCCUCUACUGCUATT-3′
siRGL3_1354	5′- ACACAGCCCUGCCGGAUAU −3′
siRGL3_1903	5′-GCGUCAGCAUCGACAAUGATT-3′
siRalGPS1_ups1	5′-GAACAAAGAUCCAAUCAGA-3′
siRalGPS1_ups3	5′-GGAUAUACCUGUGUUUAAATT-3′
siRalGPS2_231	5′-GAUUCAGCAUACCCAUCAA-3′
siRalB_107	5′-UGACGAGUUUGUAGAAGAC-3′

**Table 3:** List of reagents used in this study.

Reagent	Manufacturer	Catalogue #
Dulbecco’s modified Eagle’s medium	GE Healthcare	SH30081.01
Earle’s Balanced Salt Solution	Gibco- Life technologies	24010043
Phosphate-buffered saline	Gibco- Life technologies	10010015
L-Glutamine	Gibco- Life technologies	25030024
Penicillin-Streptomycin	Gibco- Life technologies	15140122
Fetal Bovine Serum	Biosera	FB-1003/500
Sodium Pyruvate	Gibco- Life technologies	11360070
Hygromycin B Gold	InvivoGen	ant-hg
Zeocin	InvivoGen	ant-zn
Geneticin	Gibco- Life technologies	10131035
Puromycin	InvivoGen	ant-pr
Paraformaldehyde	Electron Microscopy Sciences	15710
Glycine	Invitrogen	15527013
Triton X-100	Euromedex	2000-C
Bovine Serum Albumin	Euromedex	04-100-812-C
jetPRIME transfection buffer	Polyplus	712-60
jetPRIME transfection reagent	Polyplus	114-07
Fluoromount-G	Southern biotech	0100-01
Lipofectamine™ RNAiMAX transfection reagent	Thermo Fisher Scientific	13778150
Opti-MEM	Gibco	31985062
Protease inhibitor cocktail	Roche	0589291001

**Table 4:** List of antibodies used in this study.

Antibody	Manufacturer	Catalogue #	Dilution
Mouse monoclonal anti-RalB antibody (clone 4D1)	Sigma-Aldrich	WH0005899M4	1:200 (IF) 1:1000 (WB)
Mouse monoclonal anti-RGL2 antibody (clone 4D10)	Novus biologicals	H00005863-M02	1:200 (IF) 1:1000 (WB)
Mouse monoclonal anti-RalA antibody (clone 8)	BD Transduction Laboratories	610221	1:200 (IF)
Rabbit polyclonal anti-EEA1 antibody	Calbiochem	324610	1:200 (IF)
Goat anti-mouse secondary antibody AF488	Life technologies	A-11029	1:500 (IF)
Goat anti-mouse secondary antibody AF546	Life technologies	A-11030	1:500 (IF)
Goat anti-rabbit secondary antibody FITC	Invitrogen	F-2765	1:500 (IF)
Rabbit polyclonal anti-LC3B antibody	Cell Signaling	2775	1:1000 (WB)
Mouse monoclonal anti-β-Actin antibody (clone AC-74)	Sigma-Aldrich	A2228	1:10000 (WB)
Goat anti-mouse HRP-conjugated secondary antibody	Jackson immuno research laboratories	115-035-003	1:12000 (WB)
Goat anti-rabbit HRP-conjugated secondary antibody	Jackson immuno research laboratories	111-035-114	1:12000 (WB)
IRDye-conjugated secondary antibodies	LI-COR Biosciences	NA	1:12000 (WB)

### Cell culture, transfections, RT-qPCR

HEK-HT and HEK-HT-H-RasV12 cells^31,32^ were obtained from Chris Counter laboratory. Cells were cultured at 37 °C with 5% CO2 in DMEM media containing 10% FBS, 1% Sodium Pyruvate, 1% Penicillin-Streptomycin and 1% L-Glutamine. Hygromycin (100 μg/mL) and Geneticin (400 μg/mL) were added in HEK-HT cell culture media. Hygromycin, Geneticin, and Zeocin (300 µg/µL) were added in HEK-HT-H-RasV12 cell culture media. Puromycin (0.5 μg/mL) was added in culture media of cells stably expressing iRFP-LC3 constructed via lentivirus infection. To induce autophagy, cells were starved for 4 hrs in EBSS serum and amino acid free media.

Cells were transfected with plasmid DNAs using jetPRIME transfection reagent according to manufacturer’s protocol. Cells were seeded in 6-well plates at 0.5 × 10^6^ cells per well; 24 hrs later, transfection mix containing 2 μg plasmid DNA, 200 μL of jetPRIME buffer, and 4 μL of jetPRIME reagent were added dropwise to the cells; medium was changed 4 hrs later. Cells were used for experiments 24 hrs after DNA transfection.

Cells were transfected with siRNAs using Lipofectamine™ RNAiMAX transfection reagent according to manufacturer’s protocol. Typically cells were seeded in 6-well plate at 2.5 × 10^5^ cells per well; 24 hrs later, the transfection mix containing 3 uL Lipofectamine™ RNAiMAX, and 10 nM of siRNA, in 500 µl opti-MEM, was prepared, incubated 20 min at room temperature, and added to each well, followed by 2 mL culture medium per well; medium was changed 24 hrs later and experiments were performed 48 or 72 hrs after siRNA transfection.

RT-qPCR protocols and primers for the six RalGEFs have been described previously^45^.

### Western blotting

Cell were lysed at 4 °C in lysis buffer (50 mM Tris-HCl pH 8.0, 150 mM NaCl, 0.5% sodium deoxycholate, 0.1% SDS, 0.5% Triton X-100, freshly supplemented with 1 mM DTT and protease inhibitor cocktail). Laemmli buffer was then added to the whole cell lysate and boiled at 95 °C for 10 min. Whole cell lysate was loaded on Novex NuPAGE 10% Bis-Tris (# NP0301BOX) and transferred on 0.45 μm nitrocellulose membranes. Blocking was done with 5% BSA in TBS-tween for 30 min at RT. Membranes were incubated with primary antibodies overnight at 4 °C, washed, and then incubated with secondary antibodies for 1 hr at RT. Detection was performed alternatively with enhanced chemiluminescence method (Western Lightning Plus-ECL, PerkinElmer) when using HRP-conjugated secondary antibodies or with the LICOR Odyssey Infrared Imaging System (LI-COR Biosciences) when using IRDye-conjugated secondary antibodies.

### Immunofluorescence

Cells were cultured on coverslips for 24 hours, washed trice with Phosphate-buffered saline (PBS), and then fixed using 4% paraformaldehyde in PBS at room temperature (RT) for 10 minutes followed by 3 washes in PBS. Cells were incubated for 3 minutes at RT in 1 M Glycine solution to avoid quenching followed by 3 washes in PBS. Cells were permeabilized using 0.1% of Triton-100X in PBS for 10 min at RT and washed trice with PBS. To block not-specific binding sites, cells were incubated at RT for 45 min in PBS with 4% FBS and 1% BSA (blocking buffer). Cells were incubated with primary antibody diluted in blocking buffer for 1 hr at RT. Cells were washed with PBS and incubated with secondary antibody diluted in blocking buffer for 1 hr at RT followed by 3 washes in PBS. Coverslips were dried and then mounted on slide using Fluoromount-G mounting media.

Immunofluorescent samples were imaged using a laser scanning confocal microscope LSM 710 NLO (Zeiss, Jena, Germany) equipped with 63×/1.4NA oil-immersion objective (Zeiss). An Argon 488 laser 40 mW (Green), DPSS laser 561 20 mW (Red), and Helium-Neon 633 (Far red) were used to excite Alexa Fluor 488, Alexa Fluor 561, and iRFP fluorophores respectively. Samples were visualized on standard photomultiplier tube (PMT) detector.

### FRET measurements

Cells were plated on 35-mm glass bottom dishes (Mattek, Cat. No. # P35G-0.170-14-C) and were transiently transfected with a plasmid expressing a RalB FRET biosensor^33^. Images were acquired using a Leica DMIRE2 inverted microscope equipped with 63x objective with 1.32 NA. Samples were excited in CFP channel (430 nm) and both CFP emission (480 nm) and FRET emission (530 nm) were recorded. Binning of 2 × 2 for early endosomes, recycling endosomes and 3 × 3 binning for autophagosomes were used. FRET analysis was carried out using ImageJ software. First, the background was subtracted using a region outside the cell. Then the image was segmented manually and FRET/CFP ratio was depicted using ratiometric image with fire color code. Calibration bar represents the FRET ratio. Whole segmented cells were used to represent the FRET ratio in entire cell. A 1.67 μm-wide band from the cell periphery was segmented to measure the FRET ratio at the cell edges. To measure FRET in endomembrane compartments, the endomembrane compartments were first segmented using co-transfected specific markers (RFP-Rab5A, mCherry-Rab11, and mCherry-LC3), then FRET ratio were measured in these regions.

### Image processing with endomapper plugin

The Endomapper method was developed to be used with ImageJ software^46^ (https://imagej.nih.gov/ij/). The plugin can be downloaded from: https://github.com/mformanu9/Endomapper.git

The execution of Endomapper plugin pops up a window that asks user to enter some parameters such as pixel size, approximate size of endomembrane compartments, numbers of randomized compartments, input and out folder path. The input image for Endomapper is a dual channel image in which one channel corresponds to protein of interest and the other channel to endomembrane compartments. A region of interest (ROI) is manually selected by the user of dimension 300 × 300 pixel, aiming at including most of the endomembrane compartments. To segment the endomembrane compartments Gaussian blur filter is used followed by thresholding. Morphological operations such as fill holes and close are used to refine image. Subsequently, analyze particle tool is used to discard the unwanted objects with very small size. Once the endomembrane compartments are identified, the mean intensity in the ROI in the protein channel is measured. Then the mean intensity of the protein of interest is measured at each segmented compartment. The results of these measurements (area, mean intensity, standard deviation) are saved in a text file in the destination folder. In parallel, user defined number of randomized pseudo-compartments are created, with a size comparable to that of the endomembrane compartments under investigation. The results of these measurements (area, mean intensity, standard deviation) are saved in another text file in the destination folder. Finally, the mean intensity of the protein of interest at each endomembrane compartment is compared to its mean intensity in the entire ROI. To qualify as an endomembrane compartment positive for the protein of interest, the mean intensity of the endomembrane compartment must be higher than the sum of mean and standard deviation (mean + 1 fold SD) of protein intensity in the entire ROI.

### Statistical analysis

Results are shown as mean ± standard error of the mean (SEM). Graphs were created and statistical analysis was performed using Graphpad Prism (v5.0). All the tests were performed using Mann Whitney test. P value less than 0.05 or 0.01 were considered significant, depending on the experiments, as indicated in legends.

## Supplementary information

Supplementary figures

## Data Availability

No datasets were generated or analyzed during the current study.
