# Publisher Correction: Localization of RalB signaling at endomembrane compartments and its modulation by autophagy

**DOI:** 10.1038/s41598-019-48085-5

**Published:** 2019-08-08

**Authors:** Manish Kumar Singh, Alexandre P. J. Martin, Carine Joffre, Giulia Zago, Jacques Camonis, Mathieu Coppey, Maria Carla Parrini

**Affiliations:** 1 Institut Curie, Centre de Recherche, Paris Sciences et Lettres Research University, 75005 Paris, France; 2 ART group, Inserm U830, 75005 Paris, France; 3 Centre de Recherches en Cancérologie de Toulouse (CRCT), Inserm UMR1037, Toulouse, France; 4 LOCCO group, UMR168, 75005 Paris, France

Correction to: *Scientific Reports* 10.1038/s41598-019-45443-1, published online 20 June 2019

In the original version of this Article, the author Manish Kumar Singh was incorrectly indexed. This error has now been corrected.

