# Supplementary material for: Localization of RalB signaling at endomembrane compartments and its modulation by autophagy
Source: Sci Rep. 2019 Jun 20;9:8910. doi: 10.1038/s41598-019-45443-1 (PMC6586930; doi:10.1038/s41598-019-45443-1)
Supplement: Supplementary file 1 — Supplementary figures [file 41598_2019_45443_MOESM1_ESM.pdf]

Localization of RalB signaling at endomembrane compartments and its modulation by autophagy

Manish Kumar Singh[1,2], Alexandre PJ Martin[1,2], Carine Joffre[3], Giulia Zago[1,2], Jacques Camonis[1,2], Mathieu Coppey[1,4], Maria Carla Parrini[1,2,#]

[1] Institut Curie, Centre de Recherche, Paris Sciences et Lettres Research University, 75005 Paris, France; [2] ART group, Inserm U830, 75005 Paris, France; [3] Centre de Recherches en Cancérologie de Toulouse (CRCT), Inserm UMR1037, Toulouse, France; [4] LOCCO group, UMR168, 75005 Paris, France.

[#] Corresponding author: maria-carla.parrini@curie.fr

Supplementary figures

A

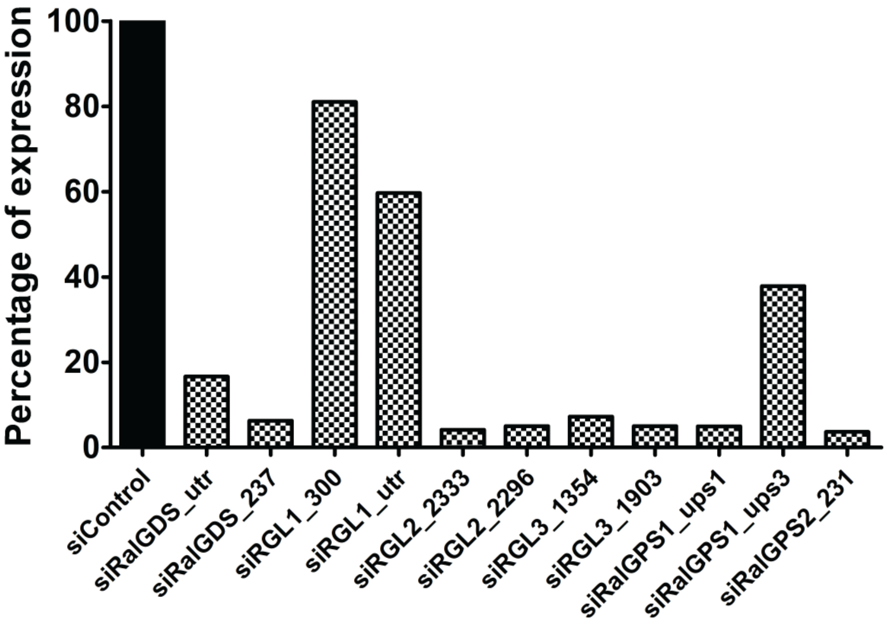

B

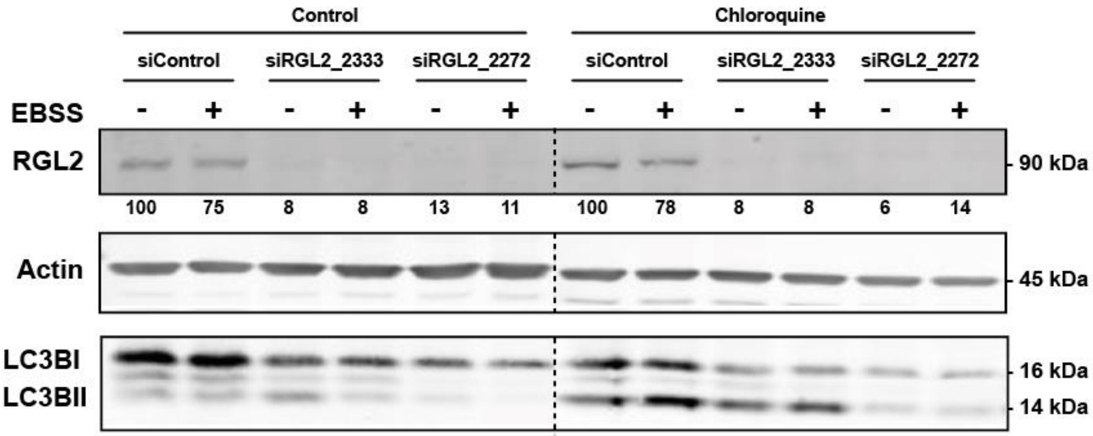

Figure S1. Validation of depletions by siRNAs.

(A) Validation of RalGEFs' depletion for autophagy screen in Hela cells. Depletion of mRNA levels is quantified by RT-qPCR for the six RALGEFs, in one experiment (n=1). Note that even if the depletion of RGL1 mRNA is modest, it is sufficient to impact on autophagy (Figure 1A). Depletions could not be validated by western-blots because specific, sensitive antibodies do not exist for RALGEFs (with the exception of RGL2).

(B) RGL2 depletions and LC3 conversion assay in HEK-HT cells. Representative western blots for RGL2, actin, LC3 from cell lysates of HEK-HT cells prepared 72 hrs after transfection with the indicated siRNAs. Quantifications of RGL2 protein depletion, normalized for siControl condition (=100), are shown below the WB. The vertical dotted lines indicate positions where gel images were cut in order to juxtapose non-adjacent lanes coming from the same gels.

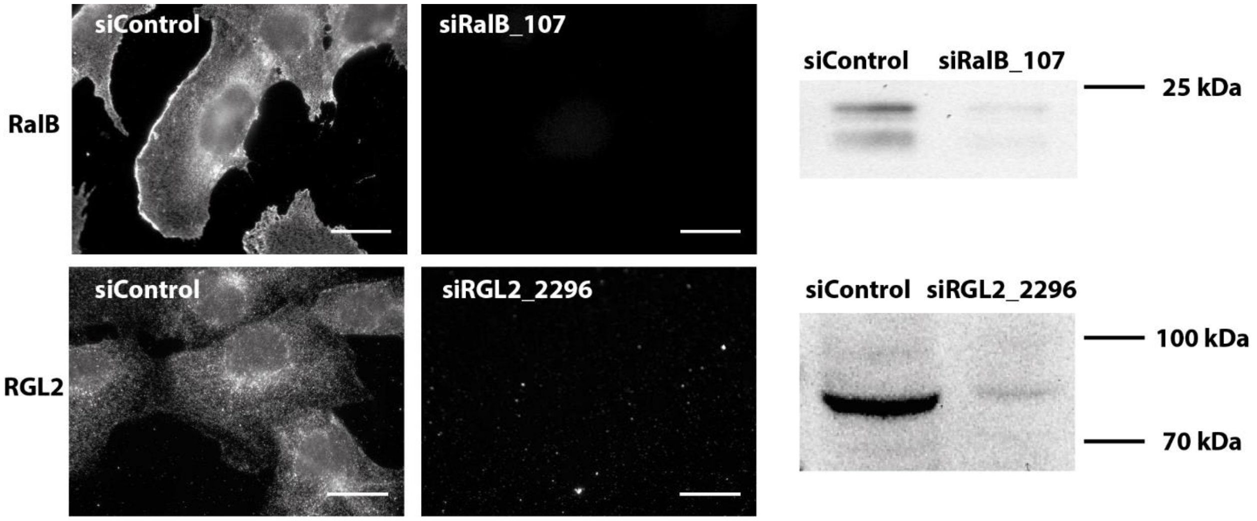

Figure S2. Validation of RGL2 and RalB antibodies for immunofluorescence (IF) applications.

Cell were transfected using non-targeting siControl, siRNA against RalB or siRNA against RGL2. Western blots show efficient protein depletions. Upon depletion of RalB or RGL2, there is a dramatic decrease of RalB or RGL2 IF signal, respectively, demonstrating the specificity of these antibodies and the reliability of the IF quantifications presented in this work. Scale bars are 20 µm.

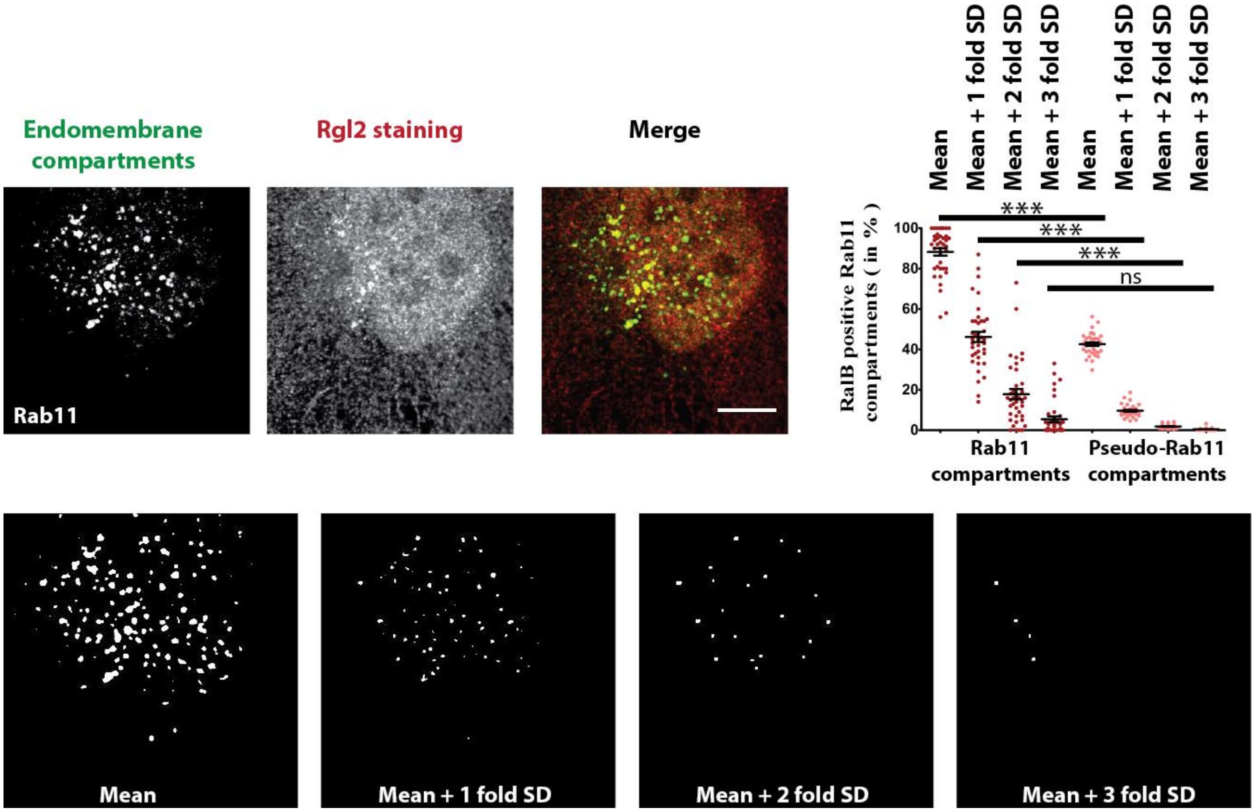

Figure S3. Comparison of thresholds to define positive compartments.
HEK-HT-H-RasV12 cells were transfected with GFP-Rab11 (marker of recycling endosomes), fixed and immunostained for RGL2. Representative confocal cross sections are shown (top left). The percentage (%) of Rab11 compartments positive for RGL2 as compared to the control pseudo-Rab11 compartments, is calculate using different thresholds: Mean (= mean RGL2 fluorescence intensity in segmented compartments), Mean + 1 fold Standard Deviation (SD), Mean + 2 folds SD, and Mean + 3 folds SD (top right). Bottom row depicts the loss of detection of recycling compartments with increase in threshold. Graph represents mean ± SEM of n = 39 cells from 4 independent experiments. For statistics Mann Whitney test was used. ** p value <0.01, *** p value <0.001. Scale bars are 10 µm.

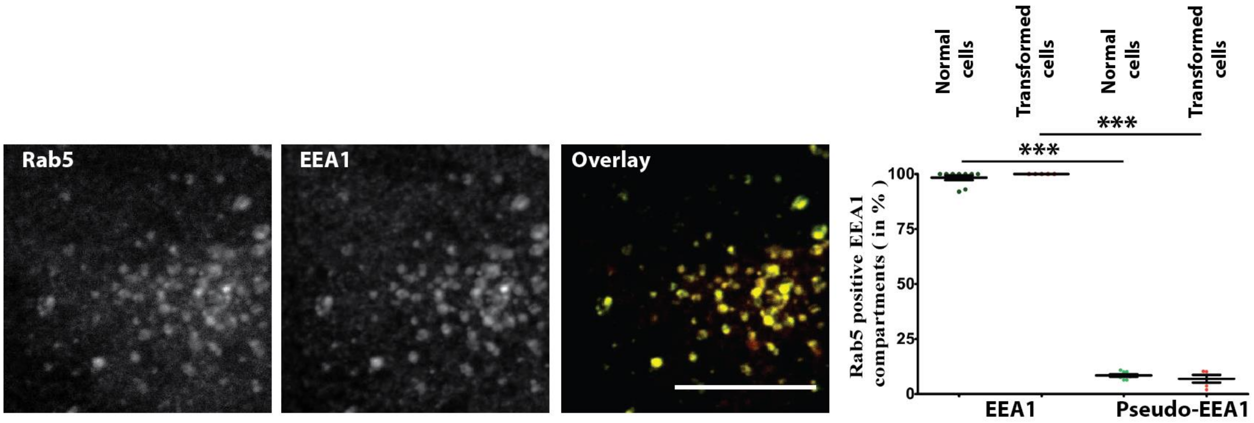

Figure S4. Validation of Endomapper analysis method using two different markers for the same compartment (early endosomes).

HEK-HT (Normal cells) and HEK-HT-H-RasV12 (Transformed cells) were transfected with Rab5 (marker of early endosomes) and then stained with antibody against early endosomes (EEA1). Each dot corresponds to one cell. Quantification graph shows that virtually 100% of EEA1 compartments are positive for Rab5. Pseudo-EEA1 compartments are used as negative controls. Graph represents mean ± standard deviation of n = 5 to 10 cells. For statistics Mann Whitney test was used. ** p value < 0.01, *** p value < 0.001. Scale bars are 10 µm.

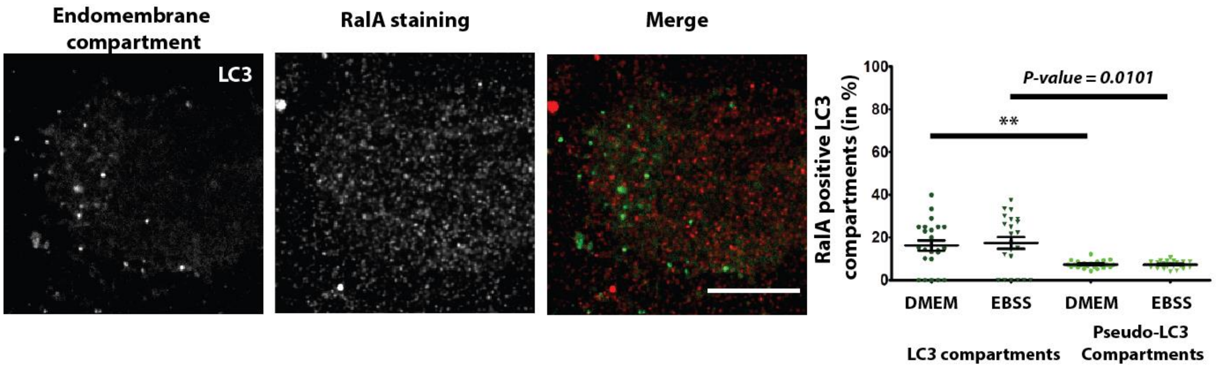

Figure S5. Localization of RalA at autophagosomes.

HEK-HT (Normal cells) were incubated in basal (DMEM) and starvation (EBSS, 4hrs) conditions, then fixed and imaged for endogenous RalA (IF anti-RalA, represented in red in overlay images), together with Cherry-LC3 (autophagosome marker, represented in green). Representative confocal cross sections are shown (left). Localization was calculated as percentage (%) of the LC3 compartments positive for RalA, as compared to control pseudo-compartments (right). Each dot corresponds to one cell. Graph represents mean ± SEM of $n \geq 22$ cells per condition from 1 experiment. For statistics Mann Whitney test was used. ** p value <0.01, *** p value <0.001. Scale bars are 10 µm.
